# Optogenetic Stimulation of Ca2+ Influx via Channelrhodopsin CapChR2 in Schwann Cells Promotes Neurite Outgrowth in Co-cultured PC12 Cells: A Neuronal Model

**DOI:** 10.7759/cureus.90023

**Published:** 2025-08-13

**Authors:** Moe Tsutsumi, Kaori Sato-Numata, Chawapun Suttinont, Tomohiro Numata

**Affiliations:** 1 Mirai Technology Institute, Shiseido Co. Ltd., Yokohama, JPN; 2 Department of Integrative Physiology, Akita University Graduate School of Medicine, Akita, JPN

**Keywords:** ca2+ influx, channelrhodopsin, co-culture system, neurite outgrowth, optogenetics, pc12 cell, peripheral nerve regeneration, schwann cell

## Abstract

Introduction: Schwann cells (SCs) are essential players in peripheral nerve regeneration, contributing to both myelination and neuronal support via paracrine and contact-mediated mechanisms. Ca^2+^ influx is a critical regulator of cellular activation with diverse cells; however, the potential of optogenetic manipulation of Ca^2+^ signals in SCs to influence neuronal growth has not been thoroughly investigated. This study aimed to evaluate whether light-induced Ca^2+^ entry through a Ca^2+^-permeable channelrhodopsin (CapChR2) in SCs could enhance neurite outgrowth in co-cultured PC12 cells.

Methods: Immortalized Fischer rat SCs (IFRS1) were genetically modified to express CapChR2 and exposed to blue light. Intracellular Ca^2+^ dynamics were monitored using fura-2-based ratiometric imaging. Whole-cell patch-clamp recordings were used to assess light-gated cation influx through CapChR2 and characterize photocurrent properties. For functional assays, PC12 cells were co-cultured with IFRS1 under four different conditions: with or without light stimulation, and with or without CapChR2 expression. Neurite outgrowth was assessed by calculating the percentage of cells with neurites longer than ten micrometers and by measuring the average neurite length.

Results: CapChR2-expressing IFRS1 showed an increase in intracellular Ca^2+^ levels upon light stimulation (ΔRatio: 0.13 ± 0.05) compared to dark controls (0.04 ± 0.02). In contrast, vector-transfected cells did not respond to light stimulation. Whole-cell patch-clamp recordings confirmed light-evoked inwardly rectifying photocurrents in CapChR2-expressing cells, whereas no photocurrent was detected in vector-transfected control cells. Ion selectivity analysis indicated variable Ca^2+^ permeability with the duration of light exposure, and the PCa^2+^/PNa^+^ ratio was estimated to be approximately 1.2 at 10 s and 0.7 at 30 s. In functional co-culture assays, PC12 cells grown with CapChR2-expressing IFRS1 displayed a significantly higher proportion of neurite-bearing cells (59.6-73.6 %) under both with and without light stimulation, compared to control conditions (41.4-47.3%). Moreover, a significant increase in neurite length was observed only in PC12 cells co-cultured with CapChR2-expressing IFRS1 under light-stimulated conditions.

Conclusion: Optogenetic activation of CapChR2-expressing IFRS1 successfully induced light-dependent Ca^2+^ influx and enhanced their capacity to promote neurite outgrowth in co-cultured PC12 cells. These results highlight the utility of glial optogenetic modulation as a novel and controllable approach to facilitate axonal regeneration. This strategy may hold therapeutic potential for the treatment of peripheral nerve injuries and demyelinating disorders by enhancing the regenerative microenvironment through targeted glial activation.

## Introduction

Peripheral nerve regeneration relies on the supportive roles of Schwann cells (SCs), which promote axonal growth, guide regenerating fibers, and form myelin sheaths. In the injured nervous system, SCs become activated and undergo morphological and functional changes, including increased proliferation, secretion of neurotrophic factors, and upregulation of surface molecules that influence axonal regrowth [1-3]. Among the key intracellular signals that regulate these processes, Ca^2+^ influx is a critical trigger for SCs activation and modulation of their phenotype, influencing processes such as proliferation, neurotrophic factor secretion, and support for nerve regeneration [4,5].

Recent advances in optogenetics provide powerful tools for manipulating intracellular signals with high spatiotemporal precision. Channelrhodopsins, a class of light-sensitive ion channels, can be used to regulate Ca^2+^ entry in targeted cell populations [6]. While optogenetic approaches have been widely applied to neurons, their application to glial cells such as SCs is a promising yet underexplored area of research [7,8]. Previous studies have shown that optogenetic stimulation of SCs can enhance their proliferation and promote differentiation into myelinating phenotypes, even in the absence of neurons [9].

Moreover, optogenetic activation of SCs has been shown to influence neuronal functions in co-culture systems [7,10]. These findings emphasize the growing recognition of the importance of glia-neuron communication in shaping neural development, plasticity, and regeneration. Despite this, few studies have directly addressed how optogenetically induced Ca^2+^ influx in SCs affects neurite outgrowth in adjacent neurons. Understanding this interaction is essential for developing glia-targeted strategies for nerve repair and regeneration.

In this study, we investigated whether optogenetic induction of Ca^2+^ influx in SCs via a Ca^2+^-permeable channelrhodopsin (CapChR2) could promote neurite outgrowth in co-cultured PC12 cells, a model of sympathetic neurons. We hypothesized that light-driven Ca^2+^ entry in SCs enhances their supportive function for neurite extension, offering insights into the potential utility of optogenetics in glia-mediated neural repair.

## Materials and methods

Cell culture

Immortalized Fischer rat Schwann cells (IFRS1) were obtained from Cosmo Bio Co., Ltd. (Tokyo, Japan). Cells were maintained in low-glucose Dulbecco's Modified Eagle Medium (DMEM; FUJIFILM Wako, Osaka, Japan) supplemented with 10% fetal bovine serum (FBS; Hyclone Laboratories, UT, USA), 1% GlutaMAX (Thermo Fisher Scientific, Waltham, MA, USA), and Penicillin-Streptomycin Mixed Solution (Stabilized) (Nacalai Tesque, Inc., Kyoto, Japan). For cell proliferation, recombinant human heregulin-β1 (Thermo Fisher Scientific) and forskolin (Sigma-Aldrich, St. Louis, MO, USA) were added to the cell culture medium. Cells were cultured at 37°C in a humidified atmosphere containing 5% CO_2_ and passaged every two to three days, and cells were used for experiments at passages 3-10. For substrate coating, culture vessels were pre-coated with poly-D-lysine (PDL; Thermo Fisher Scientific) and then laminin (FUJIFILM Wako) was added before cell seeding. Experiments were typically conducted when cells reached 70-80% confluence, usually one day after seeding. Transfections were performed on the following day (day one), and functional assays, including Ca^2+^ imaging, patch-clamp recordings, or co-culture with neurons, were conducted on 24-48 hours post-transfection, depending on the specific protocol. PC12 cells were maintained in high-glucose DMEM (FUJIFILM Wako) supplemented with 5% heat-inactivated horse serum (Thermo Fisher Scientific), 5% FBS, and antibiotics as described above. Cells were passaged every three to four days and used for experiments between passages 5 and 20. For neurite outgrowth experiments, cells were seeded at ~50-60% confluence on PDL-coated coverslips and induced to differentiate in high-glucose DMEM (4.5 g/L D-glucose; Thermo Fisher Scientific) without nerve growth factor to allow assessment of SC-mediated effects. PC12 cells were employed as a neuronal model based on their established capacity to extend neurite-like processes in response to glial-derived cues, making them a widely used surrogate for sympathetic neurons in neuritogenesis studies. Neurite outgrowth was assessed 72 hours after seeding to evaluate SC-mediated effects on neurite extension.

Transfection of plasmid

IFRS1 was transfected one day after seeding, when they reached approximately 70-80% confluence. For each well of a six-well plate, 1 μg of CapChR2 pmScarlet-N1 plasmid DNA (Addgene plasmid #188032) [11] and 2 μL of Lipofectamine® 2000 (Thermo Fisher Scientific) were diluted in 50 μL of Opti-MEM® (Thermo Fisher Scientific) and incubated separately for 5 min at room temperature. The two solutions were then combined, gently mixed, and incubated for an additional 20 min to allow complex formation. The resulting DNA-lipid complexes were added dropwise to the cells and incubated at 37°C in a humidified atmosphere containing 5% CO_2_ for 24-48 hours. Transfection efficiency was confirmed by fluorescence microscopy for the pmScarlet reporter at 24-48 hours post-transfection. For electrophysiological validation of CapChR2 function, HEK293T cells were used due to their high transfection efficiency and suitability for whole-cell patch-clamp recordings. HEK293T cells were maintained in standard DMEM supplemented with 10% FBS at 37°C in a humidified 5% CO₂ atmosphere. These cells were transfected under the same conditions as IFRS1 cells and recorded 24-48 hours post-transfection to assess light-induced currents. This validation procedure is consistent with the methods described in the “Electrophysiology” section below and was used to confirm CapChR2 channel activity before functional assays in IFRS1 or PC12 co-culture experiments.

Measurement of intracellular Ca^2+^ changes

Intracellular cytoplasmic free calcium concentration ((Ca^2+^)_i_) in IFRS1 was measured using the ratiometric fluorescent Ca^2+^ indicator fura-2/AM (Dojindo Laboratories, Kumamoto, Japan). Blue light stimulation (400-450 nm, 0.5 mW/cm^2^, 20 min) was applied via a Hayashi Lepic xenon light source (Luminar Ace LA-410UV) equipped with a 400-450 nm bandpass filter. Cells were seeded onto PDL-coated 13-mm glass coverslips (Matsunami Glass, Tokyo, Japan) and incubated in DMEM solution containing 5 μM fura-2/AM for 30 min at 37°C. After incubation, the coverslips were transferred to a 0.3 mL experimental chamber mounted on an inverted microscope (IX81, Olympus, Tokyo, Japan). Fura-2 was excited alternately at 340 nm and 380 nm using a Polychrome IV monochromator system (Till Photonics, Martinsried, Germany) equipped with a xenon arc lamp. Emitted fluorescence was collected through a 475 nm long-pass emission filter and imaged using a digital cooled CCD camera (Hamamatsu Photonics, Shizuoka, Japan) mounted on an inverted fluorescence microscope equipped with a 20× objective lens. Images were acquired every 30 s, and the fluorescence intensity ratio (F340/F380) was calculated using MetaFluor software (Molecular Devices, San Jose, CA, USA). Cells expressing CapChR2 were identified based on pmScarlet fluorescence. For each experiment, only cells showing a stable baseline fluorescence ratio for at least one minute before stimulation was selected. Blue light stimulation (470 nm, 0.5 mW/cm^2^) was applied continuously for 20 min to activate CapChR2 and evoke Ca^2+^ influx. ΔRatio values were calculated by subtracting the average baseline ratio (over the one-minute pre-stimulation period) from the peak F340/F380 ratio observed during stimulation. All measurements were performed at room temperature in Tyrode's solution composed of 140 mM NaCl, 5 mM KCl, 1 mM MgCl₂, 2 mM CaCl₂, 10 mM glucose, and 10 mM HEPES, adjusted to pH 7.4 with NaOH (300 mosmol/kg-H₂O).

Electrophysiology

Whole-cell patch-clamp recordings were conducted on cells transfected with either CapChR2 pmScarlet-N1 or vector control plasmids. Recordings were performed 24-48 hours after transfection. Cells were held under voltage-clamp configuration using borosilicate glass micropipettes (resistance: 3-5 MΩ), filled with an internal solution containing: 55 mM K_2_SO_4_, 5 mM KCl, 5 mM HEPES, 0.2 mM EGTA, and 5 mM MgCl_2_ (pH adjusted to 7.2 with KOH). The standard extracellular recording solution was Tyrode's solution, as described above. For ion selectivity experiments, two different extracellular solutions were used: (1) Na^+^ solution containing 130 mM NaCl, 10 mM glucose, and 10 mM HEPES (pH 7.4 with NMDG, 320 mOsm with D-mannitol), and (2) Ca^2+^ solution in which NaCl was replaced with 65 mM CaCl_2_ to establish bi-ionic conditions. Light stimulation was applied through the objective lens using a 400-450 nm excitation filter, delivering an irradiance of up to 20 mW/cm^2^. Photocurrents were recorded at room temperature and analyzed to determine the peak amplitude and current-voltage (I-V) relationship. To evaluate Ca^2+^ selectivity, reversal potentials (E_rev_) were determined under Na^+^ and Ca^2+^ conditions. The E_rev_ were determined using ClampFit software (Molecular Devices) by analyzing I-V relationships under continuous light stimulation. Liquid junction potentials were calculated using ClampEx software (Molecular Devices), yielding estimated offsets of -8.9 mV for the Na^+^ solution and -12 mV for the Ca^2+^ solution. These values were used to correct the raw E_rev_ values before permeability analysis. The relative selectivity ratio (PCa^2+^/PNa^+^) was calculated using the Goldman-Hodgkin-Katz (GHK) equation.

Measurement of neurite outgrowth

Cell Culture and Co-culture Conditions

PC12 cells were co-cultured with IFRS1 transfected with either CapChR2_pmScarlet-N1 or mCherry-N1 vectors. Co-culture was initiated on day two post-transfection, when IFRS1 cells exhibited visible expression of the fluorescent reporter proteins. PC12 cells were seeded on PDL-coated coverslips at ~50-60% confluence, in high-glucose DMEM without nerve growth factor 1 day prior to co-culture, to allow assessment of Schwann cell-mediated effects. Both PC12 and IFRS1 cells were seeded at a density of 20,000 cells each per 3.5 cm dish. Cells were plated on PDL-coated glass coverslips and maintained in a standard growth medium optimized for IFRS1 cells. The co-cultures were divided into four groups as follows: CapChR2-expressing IFRS1 without light stimulation (dark control), CapChR2-expressing IFRS1 with light stimulation, mCherry-expressing IFRS1 without light stimulation, and mCherry-expressing IFRS1 with light stimulation. Cells were stimulated using a blue LED light source (Ex Flashlight, ExF-B, Bio Tools, Gunma, Japan) with a wavelength range of 470-485 nm and an intensity of 5 mW/cm^2^. Control groups were maintained under identical environmental conditions without light exposure. The light stimulation protocol was established based on previous studies and our preliminary experiments. The wavelength range (470-485 nm) corresponds to the optimal activation range of CapChR2 [11], and preliminary experiments indicate that 0.5 mW provides effective activation without phototoxicity. This condition delivered a cumulative energy of ~900 mJ/cm² per day, which was found in preliminary experiments to be optimal for promoting neurite outgrowth while avoiding overstimulation. Further rationale is provided in the Discussion section. The daily 30-minute stimulation period was chosen based on prior work showing neurotrophic factor release under similar conditions [12]. The three-day stimulation period was chosen based on the timeframe in which neurite outgrowth became consistently observable in our co-culture system.

Imaging and Quantification of Neurite Outgrowth

After 72 hours of co-culture, phase-contrast or fluorescence images were acquired using a Cytowatcher system or phase-contrast microscope (Evident, Tokyo, Japan). While fluorescence imaging was used to verify reporter expression, neurite outgrowth was quantified exclusively in PC12 cells, which were morphologically distinguishable from IFRS1 cells under phase-contrast microscopy. Neurite measurements were performed using CellSense software (Evident, Tokyo, Japan), and target cells were manually selected based on established morphological criteria to exclude IFRS1 cells from the analysis. Cells with neurites extending longer than 10 μm were classified as neurite-positive [13]. The percentage of neurite-positive cells was calculated relative to the total number of cells. The mean neurite length was determined by measuring all neurite-bearing cells per condition. Control groups were maintained under identical environmental conditions without light exposure and were protected from ambient light using aluminum foil-wrapped culture dishes throughout the culture period.

Statistical analysis

All data were expressed as mean ± standard error of the mean (SEM). Statistical analyses were performed using Origin Pro (OriginLab Corporation, Northampton, MA, USA). For comparisons among multiple groups, one-way analysis of variance (ANOVA) was followed by Tukey's post hoc test. A p-value of less than 0.05 was considered statistically significant. All experiments were independently repeated at least three times.

## Results

CapChR2 expression induces light-dependent Ca^2+^ influx in Schwann cells

**Figure 1:**
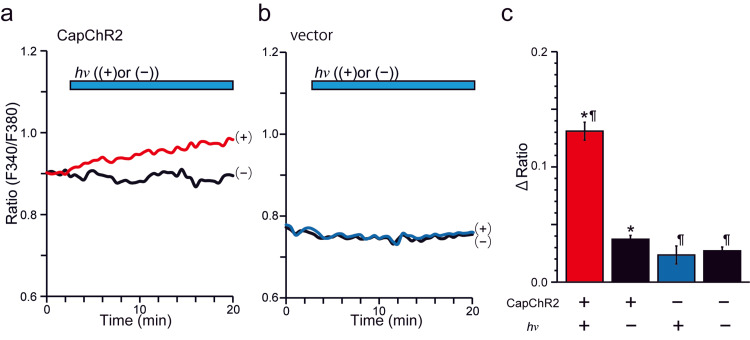
Light-evoked Ca2+ influx in CapChR2-expressing Schwann cells. (a) and (b) Representative traces of fura-2 ratiometric Ca^2+^ imaging (F340/F380) in IFRS1 transfected with CapChR2 (a) or vector control (b). Cells were subjected to blue light stimulation (470 nm, 0.5 mW/cm^2^) or kept in the dark. Light blue bars above the plots indicate the duration and presence (+) or absence (–) of light stimulation (hν). (c) Quantification of the change in fluorescence ratio (ΔRatio) after light stimulation. Data represent 33-43 cells per group from three independent biological replicates. *p < 0.05 vs. vector hv (–); ¶p < 0.05 vs. CapChR2 hv (–) (ANOVA, followed by Tukey's post hoc test). CapChR2: Ca^2+^-permeable channelrhodopsin; ANOVA: one-way analysis of variance.

To evaluate whether CapChR2 can effectively induce light-dependent Ca^2+^ influx in SCs, IFRS1 were transfected with CapChR2 and subjected to ratiometric Ca^2+^ imaging using fura-2-AM. As shown in Figure 1, upon illumination, CapChR2-expressing cells exhibited a significant increase in the F340/F380 fluorescence ratio compared to non-stimulated control cells. The average ΔRatio in the stimulated group was 0.13 ± 0.05, which was significantly greater than that observed in the dark control group (0.04 ± 0.02). In contrast, IFRS1 transfected with a vector control showed no change in fluorescence ratio upon identical light stimulation, confirming that the observed Ca^2+^ influx was mediated explicitly by CapChR2 expression. Additionally, CapChR2-expressing cells demonstrated a higher baseline fluorescence ratio before stimulation (0.90 ± 0.04; *p > 0.05, n = 35-43) compared to vector controls (0.76 ± 0.05), suggesting the presence of basal channel activity even in the absence of photostimulation. These findings support that CapChR2 enables effective and specific optical control of intracellular Ca^2+^ dynamics in IFRS1.

Light-evoked cation currents and Ca^2+^ permeability of CapChR2

**Figure 2:**
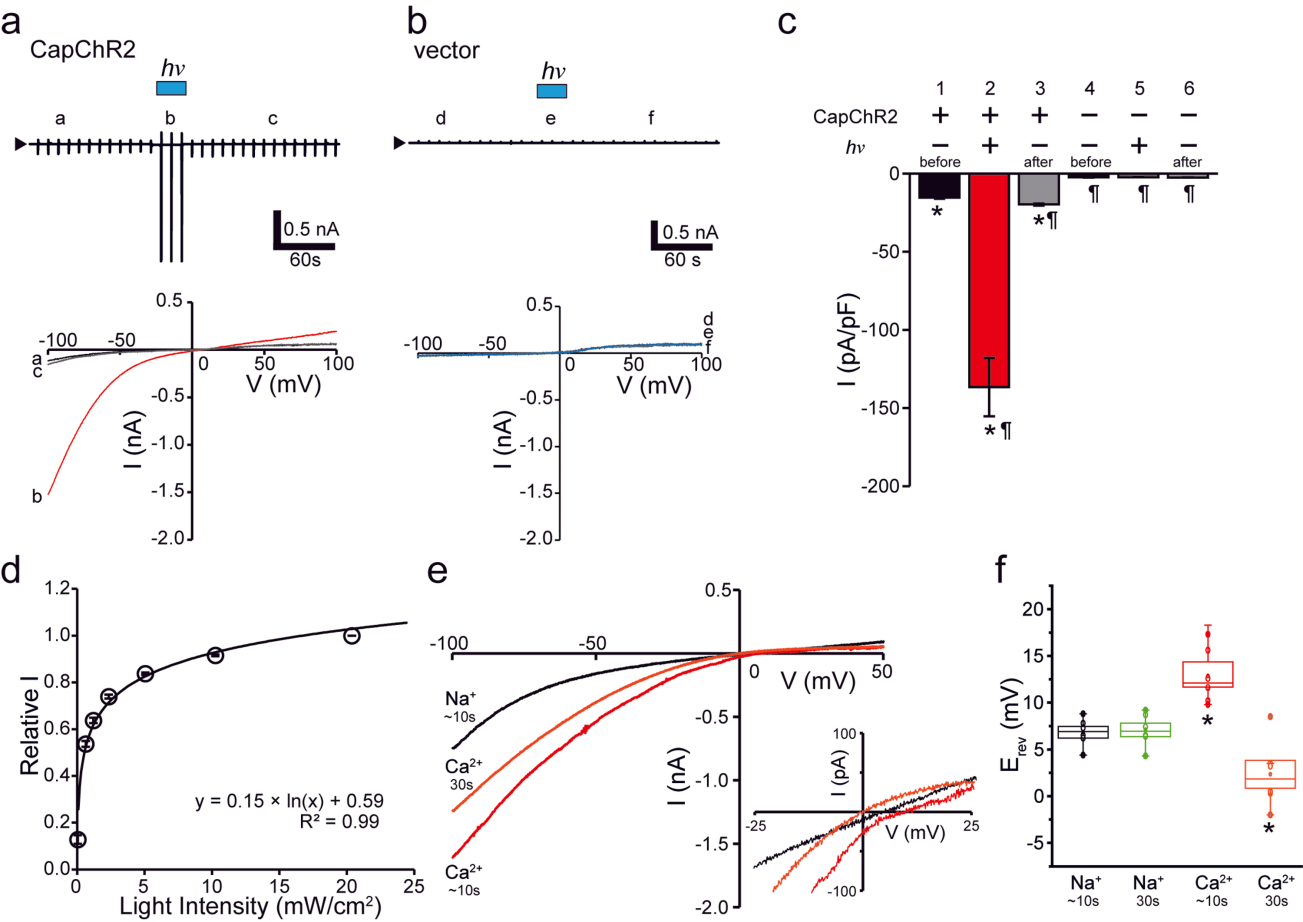
Light-evoked whole-cell currents and Ca2+ permeability of CapChR2. (a, b) Top: Representative whole-cell patch-clamp recordings from cells transfected with CapChR2 (a) or vector control (b). Light stimulation (hv: 400-450 nm, 20 mW/cm^2^) is indicated by the light blue bar above the traces. Bottom: Current-voltage (I-V) relationships were obtained before (a) and (d), during (b) and (e), and after (c) and (f) hv. (c) Quantification of light-induced current density (pA/pF) before and after illumination in CapChR2- and vector-transfected cells. Bars 1-6 correspond to the following experimental conditions: 1. CapChR2, before hv; 2. CapChR2, during hv; 3. CapChR2, after hv; 4. Vector, before hv; 5. Vector, during hv; 6. Vector, after hv. Data represent six cells per condition from three independent biological replicates. *p<0.05 vs. 4; ¶p<0.05 vs. 1 (ANOVA, followed by Tukey’s post hoc test). (d) Light intensity–response curve showing normalized peak current amplitudes as a function of irradiance (mW/cm^2^). Data are from 6 cells across three independent experiments. The current response, normalized to the value at 20 mW/cm^2^, exhibited a logarithmic relationship with hv. (e) I-V curves recorded by voltage ramp protocols in extracellular solutions containing either Na^+^ (black, ~10s), Ca^2+^ (red, ~10s), and Ca^2+^ (orange, 30s). The inset shows a magnified view of the I-V relationship near the reversal potential. (f) Box plots of reversal potentials (E_rev_) measured from light-induced whole-cell currents under Na^+^ and Ca^2+^ conditions at 10s and 30s after application. A depolarizing shift in Erev was observed in the Ca^2+^ solution at 10s, indicating Ca^2+^ permeability of CapChR2, with an estimated PCa^2+^/PNa^+^ of approximately 1.2 at 10s and 0.7 at 30s. Data represent 6 cells per condition from three independent experiments. Statistical analysis was performed using ANOVA, followed by Tukey's post hoc test. *p<0.05 indicates a statistically significant difference compared to the Na^+^~10s group. Student's t-test after confirming equal variance by F-test.

To further characterize the functional properties of CapChR2, whole-cell patch-clamp recordings were performed on cells transfected with either CapChR2 or a control vector. Membrane currents were evoked by applying voltage ramp pulses (from -100 mV to +100 mV at 1.3 mV/ms) every 10 s from a holding potential of 0 mV. Upon light illumination, CapChR2-expressing cells exhibited inwardly rectifying photocurrents, whereas vector-transfected control cells showed no light-evoked response (Figures 2a-2c). Notably, the photocurrents were initiated immediately at the onset of illumination and declined upon cessation of light exposure. However, residual currents persisted after the termination of light (Figures 2a-2c), suggesting that the channel activity is not only clearly light-dependent but also partially retained after activation.

To assess light intensity-response characteristics, the peak current amplitude was plotted against irradiance, revealing a logarithmic relationship (Figure 2d). The fitted dose-response curve estimated a half-maximal effective irradiance (EC_50_) of approximately 0.76 mW/cm^2^, assuming a normalized maximum response of 1 and a minimum of 0.1.

We next investigated the ion selectivity of the light-induced currents by measuring E_rev_ under bi-ionic conditions in which either Na^+^ or Ca^2+^ served as the predominant extracellular cation. In Na^+^-containing solution, the E_rev_ remained relatively stable (6.8 mV at 10 s and 7.1 mV at 30 s post-stimulation) (Figures 2e, 2f; Na^+^). In contrast, under Ca^2+^-containing conditions, the E_rev_ exhibited a depolarizing shift at 10 s (12.9 mV) (Figure 2e; red traces, Figure 2f; Ca^2+^-10 s), followed by a marked shift toward hyperpolarization at 30 s (2.3 mV) (Figure 2e; orange traces, Figure 2f; Ca^2+^-30 s). These dynamic changes yielded estimated permeability ratios (PCa^2+^/PNa^+^) of approximately 1.2 at 10 s and 0.7 at 30 s, suggesting a time-dependent modulation of Ca^2+^ permeability that remained stable for at least five minutes during extended recordings.

Collectively, these findings indicate that CapChR2 operates as a light-gated, Ca^2+^-permeable cation channel, exhibiting conductance characteristics of both a distinct light-induced current component and a small persistent component following light illumination. Its dynamic ion permeability profile highlights its potential as a versatile optogenetic tool for modulating intracellular Ca^2+^ signaling in SCs.

Optogenetically stimulated Schwann cells enhance neurite outgrowth in co-cultured PC12 cells

To evaluate the effect of optogenetically induced Ca^2+^ influx in SCs on neuronal differentiation, PC12 cells were co-cultured with IFRS1 under four experimental conditions: (1) CapChR2-transfected IFRS1 without light stimulation, (2) CapChR2-transfected IFRS1 with light stimulation (470 nm, 0.5 mW/cm^2^), (3) vector-transfected IFRS1 with light stimulation, and (4) vector-transfected IFRS1 without light stimulation. After 72 hours of co-culture, the extent of neurite outgrowth was assessed by calculating the percentage of PC12 cells bearing neurites longer than 10 μm.

**Figure 3:**
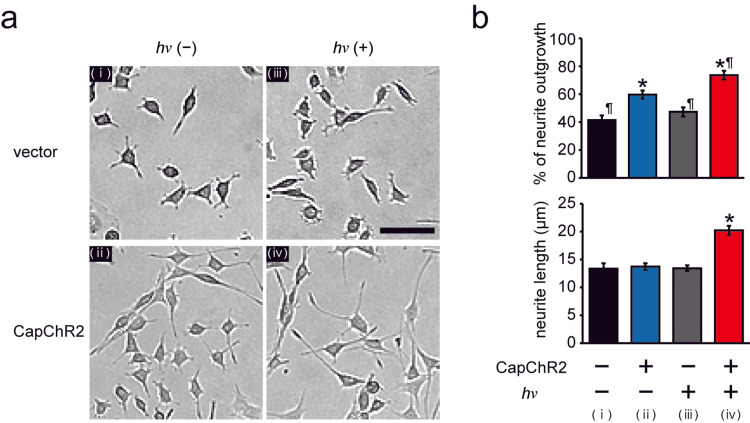
Optogenetically activated Schwann cells promote neurite outgrowth in PC12 cells. (a) Representative phase-contrast images showing neurite outgrowth in PC12 cells co-cultured with IFRS1 under four experimental conditions: (i) vector-transfected IFRS1 without light stimulation (PC12 + IFRS1 vector, hν(−)), (ii) CapChR2-transfected IFRS1 without light stimulation (PC12 + IFRS1 CapChR2, hν(−)), (iii) vector-transfected IFRS1 with light stimulation (PC12 + IFRS1 vector, hν(+)), and (iv) CapChR2-transfected IFRS1 with light stimulation (PC12 + IFRS1 CapChR2, hν(+)). Light stimulation (470 nm, 0.5 mW/cm^2^) was applied for 30 min per day over three consecutive days. Scale bar = 25 μm. (b) Top: quantification of neurite outgrowth based on the percentage of PC12 cells bearing neurites longer than 10 μm. Light-stimulated CapChR2-expressing SCs significantly increased the proportion of neurite-bearing PC12 cells compared to all control groups (n = 152–401; *p < 0.05 vs. vector hv(−), ¶p < 0.05 vs. CapChR2 hv(−)). The number of neurite-bearing cells and total analyzed PC12 cells per group were as follows: (i) vector hν(−): 63/152 cells (10 replicates), (ii) CapChR2 hν(−): 163/275 cells (18 replicates), (iii) vector hν(+): 89/188 cells (13 replicates), and (iv) CapChR2 hν(+): 292/401 cells (27 replicates). Bottom: analysis of neurite length in each group. Data represent 152–549 cells per group, analyzed across the respective numbers of independent biological replicates indicated for each condition. Statistical analysis was performed using one-way ANOVA followed by Tukey's post hoc test. *p < 0.05 vs. vector hν(−); ¶p < 0.05 vs. CapChR2 hν(−). CapChR2: Ca^2+^-permeable channelrhodopsin; ANOVA: one-way analysis of variance.

Representative phase-contrast images (Figure 3a) demonstrated a marked increase in neurite formation in PC12 cells co-cultured with light-stimulated CapChR2-expressing IFRS1. Quantitative analysis confirmed that this group exhibited a significantly higher percentage of neurite-bearing cells (73.6% ± 3.1%) compared to the dark control group (59.6% ± 3.0%), the vector-transfected IFRS1 without light (41.4% ± 3.4%), and the vector-transfected IFRS1 with light stimulation (47.3% ± 3.3%) (Figure 3b, top). Notably, even in the absence of light, co-culture with CapChR2-expressing IFRS1 resulted in a significantly greater proportion of neurite-positive cells than either vector control, suggesting a contribution from basal channel activity to neuritogenic support.

In addition, analysis of neurite length revealed a significant increase only in the group co-cultured with light-stimulated CapChR2-expressing IFRS1 (Figure 3b, bottom). No significant difference in neurite length was observed in any of the other conditions, indicating that substantial, light-dependent Ca^2+^ influx into SCs is necessary to promote axonal elongation.

Together, these findings indicate that optogenetically induced Ca^2+^ entry into SCs enhances their neurotrophic support for co-cultured PC12 cells.

## Discussion

In this study, we investigated the functional consequences of optogenetically induced Ca^2+^ influx in Schwann cells (SCs) using the Ca^2+^-permeable channelrhodopsin CapChR2. Light stimulation triggered a marked increase in intracellular Ca^2+^ concentration, as shown by fura-2-based Ca^2+^ imaging (Figure 1), and elicited inwardly rectifying photocurrents with Ca^2+^ permeability in CapChR2-expressing cells, as revealed by whole-cell patch-clamp analysis (Figure 2). These responses were absent in vector-transfected controls, confirming the specificity of CapChR2-mediated activation.

These findings align with prior reports that underscore the importance of intracellular Ca^2+^ dynamics in regulating SCs physiology, including proliferation, migration, and myelination [10,14]. Notably, Jung et al. demonstrated that optogenetic stimulation of calcium-translocating channelrhodopsin (CatCh)-expressing SCs enhanced myelination in co-culture with motor neurons. Similarly, Xu et al. suggested that optogenetic Ca^2+^ modulation promotes glial-neuron communication and metabolic support. However, few studies have directly examined the morphological consequences of light-induced Ca^2+^ influx in SCs on adjacent neuronal cells.

The present findings extend previous observations by demonstrating that photostimulation of CapChR2-expressing IFRS1 significantly enhances both neurite initiation and elongation in co-cultured PC12 cells (Figure 3). Light stimulation increased the percentage of neurite-forming cells from 59.6% in the dark control group to 73.6% ± 3.1%. Meanwhile, these two conditions were significantly higher than the percentages observed in the vector-transfected control group. Notably, a significant increase in neurite length was observed only in the light-stimulated CapChR2 group. These results suggest that optogenetically induced Ca^2+^ influx into SCs promotes the release of neurotrophic factors that facilitate neurite formation, while robust Ca^2+^ entry under photostimulation may further enhance the secretion of factors specifically involved in neurite elongation. Furthermore, as light stimulation targeted only SCs, and PC12 cells were not optogenetically modified, current-clamp recordings of neuronal firing were not applicable in this context.

The Ca^2+^ to Na^+^ permeability ratio observed in CapChR2-expressing cells was approximately 1.2, which is slightly lower than the previously reported value of 1.9 [11], yet sufficient to trigger downstream intracellular signaling in SCs. As shown in Figures 2e, 2f, Ca^2+^ permeability was highest during the initial 10 s of light exposure and gradually decreased to approximately 0.7 with prolonged stimulation. Despite this reduction, sustained photocurrent activity remained elevated above baseline (Figures 2a, 2c), suggesting continued Ca^2+^ influx beyond the peak phase. This ongoing influx likely contributes to the prolonged elevation of intracellular Ca^2+^ concentration observed in Figures 1a, 1b.

These findings support the notion that Ca^2+^ influx through CapChR2 may promote the release of neurotrophic factors or modulate SCs-neuron interactions via activation of Ca^2+^-sensitive intracellular signaling pathways, including CaMK, ERK/MAPK, and NFAT [5,15]. For example, increased intracellular Ca^2+^ has been shown to induce the secretion of brain-derived neurotrophic factor (BDNF) and glial cell line-derived neurotrophic factor (GDNF) from SCs, all of which contribute to neurite outgrowth and elongation by promoting axonal survival, growth cone dynamics, and cytoskeletal remodeling [5,16,17]. This interpretation is consistent with previous studies demonstrating the critical role of Ca^2+^-dependent signaling in regulating glial cell functions such as differentiation, myelination, and trophic support [10,18]. Unlike earlier optogenetic tools with limited Ca^2+^ selectivity, CapChR2 facilitates both an immediate, robust Ca^2+^ influx during light exposure and a sustained, moderate Ca^2+^ entry thereafter. This dual-phase Ca^2+^ influx offers precise control over SCs function for studies of neuronal regeneration. These results indicate that approximately 900-1200 mJ/cm^2^ of cumulative photostimulation, as used in our standard 0.5 mW/cm^2^ for 30 min protocol, is well suited to activate CapChR2-mediated Ca^2+^ influx and enhance neurotrophic output. In contrast, photostimulation at 5 mW/cm^2^ for 10 min (∼3000 mJ/cm^2^) failed to promote neurite elongation, despite enough total energy delivery. This suggests that higher instantaneous irradiance may exceed the optimal activation threshold for neurotrophic signaling, potentially disrupting Ca^2+^-sensitive pathways without causing overt cytotoxicity. This intensity also corresponds to the conditions used in Figure 1 and is consistent with the EC50 value (0.76 mW/cm^2^) observed in Figure 2f.

Importantly, our previous work has shown that SCs secrete neurotrophic factors such as brain-derived neurotrophic factor (BDNF) in response to mechanical stimulation [19]. In addition, vascular endothelial growth factor A (VEGFA) has been reported to promote not only angiogenesis but also glial cell proliferation and survival [20]. This raises the possibility that optogenetically triggered Ca^2+^ influx may similarly activate mechanosensitive intracellular pathways that enhance neurotrophic output. While the precise identity and regulatory mechanisms of the secreted factors under light stimulation remain to be elucidated, the current findings support the hypothesis that light-driven Ca^2+^ signaling enhances the neuro-supportive function of glial cells.

Our results further suggest that intracellular Ca^2+^ elevation in SCs may serve as a key trigger for promoting neurite outgrowth. Although the specific downstream pathways may differ depending on the source and channel of Ca^2+^ entry, the enhancement of neuronal growth by optogenetically induced Ca^2+^ influx implies that comparable effects could be achieved through pharmacological agents that elevate glial Ca^2+^. Thus, Ca^2+^ influx may represent a useful indicator in the screening of compounds aimed at stimulating glial-mediated axonal regeneration.

From a translational perspective, these findings emphasize the emerging potential of targeting glial cells, rather than neurons, as primary optogenetic intervention sites in regenerative therapies. In particular, modulating Schwann cell activity via light-gated channels may offer significant therapeutic advantages in conditions such as peripheral nerve injury and demyelinating neuropathies, where glial support is essential for axonal regrowth and remyelination [21]. The use of Ca^2+^-permeable opsins like CapChR2 enables precise, non-invasive activation of intracellular signaling pathways. Importantly, glial-targeted optogenetic stimulation has already been shown to modulate pain sensitivity [22], restore bladder control [23], and influence spinal cord repair processes [24]. When integrated with wireless or implantable photostimulation systems [25], such strategies could facilitate spatially and temporally controlled glial modulation in vivo, offering a promising platform for clinical translation.

Nevertheless, this study has several limitations. First, the use of PC12 cells as a neuron-like model, while widely accepted, does not fully replicate primary neuronal physiology. Second, the co-culture system lacks other components of the in vivo microenvironment, such as extracellular matrix dynamics or inflammatory cues, which may influence SC behavior. Third, although our results clearly show enhanced neurite outgrowth (Figure 3), the underlying mechanism-whether mediated by paracrine signaling, direct cell-cell interaction, or transcriptional changes in SCs-remains to be determined.

## Conclusions

This study establishes that optogenetic stimulation of SCs using CapChR2 leads to Ca^2+^-dependent modulation of their neurotrophic function, resulting in enhanced neurite formation and elongation in co-cultured PC12 cells. CapChR2 enables both transient and sustained Ca^2+^ influx, providing precise control over intracellular signaling.

These findings highlight the utility of glial optogenetic modulation as a promising strategy for neural regeneration. Future studies should focus on identifying Ca^2+^-regulated secreted molecules and elucidating their roles in neuronal support, with the ultimate goal of applying this technology in vivo for therapeutic purposes.

## References

[1] Min Q, Parkinson DB, Dun XP (2021). Migrating Schwann cells direct axon regeneration within the peripheral nerve bridge. Glia.

[2] Bosch-Queralt M, Fledrich R, Stassart RM (2023). Schwann cell functions in peripheral nerve development and repair. Neurobiol Dis.

[3] Wei C, Guo Y, Ci Z, Li M, Zhang Y, Zhou Y (2024). Advances of Schwann cells in peripheral nerve regeneration: from mechanism to cell therapy. Biomed Pharmacother.

[4] Heredia DJ, De Angeli C, Fedi C, Gould TW (2020). Calcium signaling in Schwann cells. Neurosci Lett.

[5] Luo B, Huang J, Lu L, Hu X, Luo Z, Li M (2014). Electrically induced brain-derived neurotrophic factor release from Schwann cells. J Neurosci Res.

[6] Rost BR, Schneider-Warme F, Schmitz D, Hegemann P (2017). Optogenetic tools for subcellular applications in neuroscience. Neuron.

[7] Hyung S, Park JH, Jung K (2023). Application of optogenetic glial cells to neuron-glial communication. Front Cell Neurosci.

[8] Eldar D, Albert S, Tatyana A, Galina S, Albert R, Yana M (2026). Optogenetic approaches for neural tissue regeneration: a review of basic optogenetic principles and target cells for therapy. Neural Regen Res.

[9] Jung K, Kim HN, Jeon NL, Hyung S (2020). Comparison of the efficacy of optogenetic stimulation of glia versus neurons in myelination. ACS Chem Neurosci.

[10] Jung K, Park JH, Kim SY, Jeon NL, Cho SR, Hyung S (2019). Optogenetic stimulation promotes Schwann cell proliferation, differentiation, and myelination in vitro. Sci Rep.

[11] Fernandez Lahore RG, Pampaloni NP, Schiewer E (2022). Calcium-permeable channelrhodopsins for the photocontrol of calcium signalling. Nat Commun.

[12] Park S, Koppes RA, Froriep UP, Jia X, Achyuta AK, McLaughlin BL, Anikeeva P (2015). Optogenetic control of nerve growth. Sci Rep.

[13] Gallo G, Letourneau PC (1999). Different contributions of microtubule dynamics and transport to the growth of axons and collateral sprouts. J Neurosci.

[14] Xu X, Mee T, Jia X (2020). New era of optogenetics: from the central to peripheral nervous system. Crit Rev Biochem Mol Biol.

[15] Castelnovo LF, Bonalume V, Melfi S, Ballabio M, Colleoni D, Magnaghi V (2017). Schwann cell development, maturation and regeneration: a focus on classic and emerging intracellular signaling pathways. Neural Regen Res.

[16] Yan X, Liu J, Ye Z (2016). CaMKII-mediated CREB phosphorylation is involved in Ca²⁺-induced BDNF mRNA transcription and neurite outgrowth promoted by electrical stimulation. PLoS One.

[17] Pérez-García MJ, Ceña V, de Pablo Y, Llovera M, Comella JX, Soler RM (2004). Glial cell line-derived neurotrophic factor increases intracellular calcium concentration: role of calcium/calmodulin in the activation of the phosphatidylinositol 3-kinase pathway. J Biol Chem.

[18] Vanoye CG, Sakakura M, Follis RM (2019). Peripheral myelin protein 22 modulates store-operated calcium channel activity, providing insights into Charcot-Marie-Tooth disease etiology. J Biol Chem.

[19] Suttinont C, Maeno K, Yano M, Sato-Numata K, Numata T, Tsutsumi M (2024). Role of Piezo2 in Schwann cell volume regulation and its impact on neurotrophic release regulation. Cell Physiol Biochem.

[20] Rosenstein JM, Krum JM, Ruhrberg C (2010). VEGF in the nervous system. Organogenesis.

[21] Jessen KR, Mirsky R (2021). The role of c-Jun and autocrine signaling loops in the control of repair Schwann cells and regeneration. Front Cell Neurosci.

[22] Nam Y, Kim JH, Kim JH (2016). Reversible induction of pain hypersensitivity following optogenetic stimulation of spinal astrocytes. Cell Rep.

[23] Zhou Z, Liao L (2021). Optogenetic neuromodulation of the urinary bladder. Neuromodulation.

[24] Ahmad A, Ashraf S, Komai S (2015). Optogenetics applications for treating spinal cord injury. Asian Spine J.

[25] Montgomery KL, Yeh AJ, Ho JS (2015). Wirelessly powered, fully internal optogenetics for brain, spinal and peripheral circuits in mice. Nat Methods.

